# Telephone follow-up based on empowerment theory to improve resilience and quality of life among patients after coronary artery stent implantation: a randomized controlled trial

**DOI:** 10.3389/fpsyt.2024.1248424

**Published:** 2024-04-25

**Authors:** Yan Hua, Mei Wang, Lu Li, Chunmian Guo, Zhaoxu Huang, Yang Li, Yue Lin, Yingjing Xiao, Chunping Ni, Aili Lv

**Affiliations:** ^1^ School of Nursing, Fourth Military Medical University, Xi’an, China; ^2^ Basic Medical Science Academy, Fourth Military Medical University, Xi’an, China; ^3^ The First Affiliated Hospital, Fourth Military Medical University, Xi’an, China; ^4^ School of Nursing, Shaanxi University of Chinese Medicine, Xianyang, China; ^5^ School of Nursing, Xi’an Jiaotong University, Xi’an, China

**Keywords:** telephone follow-up nursing, empowerment, resilience, quality of life, coronary heart disease

## Abstract

**Background:**

Coronary heart disease has a high incidence rate, a high mortality rate, a high recurrence rate, and a high medical cost. In addition, some patients need to undergo percutaneous coronary artery stent implantation (CASI), which is a kind of traumatic treatment. Patients can easily experience negative emotions such as anxiety and depression after surgery, which seriously affects quality of life.

**Objectives:**

The aim of this study was to evaluate the effectiveness of an empowerment-based telephone follow-up intervention on resilience and quality of life in patients who underwent CASI.

**Design:**

The design of the study is a randomized controlled trial.

**Methods:**

A total of 92 patients were recruited after CASI from the Internal Medicine Cardiovascular Department of a Grade A tertiary hospital in Xi’an, China. The patients were randomly divided into a control group and an intervention group. The control group performed routine care, whereas the intervention group developed a telephone follow-up program based on empowerment theory while carrying out routine care. Patients were investigated using the coronary heart disease-related knowledge questionnaire, the Connor–Davidson Resilience Scale (CD-RISC), and the 36-Item Short-Form Health Survey (SF-36) to compare the effects of the intervention before and after 1 month of intervention.

**Results:**

After a 1-month telephone follow-up intervention based on the empowerment theory for patients after CASI, the variations in knowledge related to coronary heart disease and all of its subscale scores were greater in the intervention group than in the control group. Except for the three dimensions of risk factor, induction factor, and rehabilitation-related knowledge, the variations in knowledge related to coronary heart disease and the other subscale scores were significantly different between the two groups (*p* < 0.05). The variations in resilience and scores on the three subscales in the intervention group were greater than those in the control group, and the difference between the two groups was statistically significant (*p* < 0.05). The variations in the quality of life and overall health, emotional functions, and social functions were significantly greater in the intervention group than in the control group (*p* < 0.05).

**Conclusions:**

A telephone follow-up intervention based on the empowerment theory can effectively improve the resilience and quality of life of patients after CASI. This follow-up approach can provide a theoretical basis and practical reference for hospitals and communities to carry out targeted continuing nursing for patients after CASI. The long-term effects of the intervention and its underlying mechanisms require further study.

**Clinical trial registration:**

http://www.chictr.org.cn/showproj.aspx?proj=173682, identifier ChiCTR2200064950.

## 1 Introduction

Coronary heart disease (CHD), also termed coronary artery disease (CAD) or ischemic heart disease (IHD), is a significant public health concern worldwide. American Heart Association (AHA) statistics show that approximately 19.1 million people die of cardiovascular disease every year, of which the most common is CHD, with a global incidence of approximately 240 million people (1). The China Cardiovascular Disease Report 2021 released by the China Center for Cardiovascular Disease pointed out that the number of patients with CHD in China is as high as 11 million, and the mortality rate has shown a continuous upward trend in 5 years. The high recurrence rate and high mortality rate of CHD have brought a heavy burden to individuals and the healthcare system (2). The increased burden of CHD has become the most important public health issue in China, and CHD should be prevented and controlled urgently. Percutaneous coronary artery stent implantation (CASI) is one of the main treatments for CHD (3). This method is a kind of traumatic treatment. Patients with CASI are more likely to report psychological distress, which leads to compelling demand for psychosocial support and follow-up intervention after discharge (4, 5).

In the 1990s, with the rise of positive psychology, resilience became the focus of psychological research. Research on resilience and quality of life (QOL) has been gradually applied to patients with chronic diseases such as type 2 diabetes (6), chronic heart failure (7), and cancer (8). Previous studies have shown that resilience can reduce anxiety and depression in patients, and has an important positive effect on improving QOL (9, 10).

Empowerment is a way for individuals or communities to improve their lives by increasing their power and ability (11). The World Health Organization (WHO) defines it as “a positive cooperative relationship formed to improve the health outcome and QOL of chronic diseases and the self-care strategy of patients” (12). Empowerment can strengthen patients’ self-awareness, promote their confidence, enhance their sense of responsibility for health management, and promote their physical and mental health (13). The empowerment intervention included five steps: clarifying problems, expressing emotions, setting goals, making plans, and evaluating results. In the process of intervention, nurses not only provide knowledge and skills but also explore and stimulate patients’ internal potential and promote patients’ self-determination through the use of knowledge and skills.

Empowerment-based interventions have become a major model of health education for individuals with chronic diseases (14, 15) and could substantially optimize patients’ empowerment levels and decrease psychological distress. However, empirical evidence is largely derived from studies in which a mixed population was recruited and patients who underwent CASI were not included.

In this study, a patient-centered empowerment-based intervention was conducted using a parallel-group randomized controlled trial (RCT). The core aim of the trial was to evaluate the effectiveness of an empowerment-based intervention on resilience and QOL among patients who underwent CASI, to provide references for patients’ continuous nursing after coronary stent implantation.

## 2 Methods

### 2.1 Design and setting

This study was designed as an RCT with two parallel groups. The study was conducted from June 2018 to November 2018 at a Grade A tertiary hospital in Xi’an, China.

In this study, the eligible participants were adult patients who underwent CASI, were cognitively intact, and were willing to participate. Participants with heart, liver, or kidney failure; hearing or vision impairment; obvious cognitive disabilities; or psychiatric problems were excluded because these conditions might limit them from participating in the study.

The sample size was estimated based on the following formula for clinical trials with epidemiological advantages and disadvantages: *n* = 2σ^2^×*f* (α, β)/(μ_1_–μ_2_)^2^. In the formula, *n* is the sample size required by the two groups. In this study, the setting of *n* is the same. σ is the estimated value of the two-population standard deviations, μ_1_–μ_2_ is the difference between the two means, and α and β are the false-positive error rate and the false-negative error rate, respectively (16).

Reviewing the relevant literature (17), it is expected that the psychological resilience score will increase by 10 points, μ_1_–μ_2_ ≈ 10, σ ≈ 13, α = 0.05, and β = 0.10; checking the table, we get *f*(α, β) = 10.5, and substituting it into the formula yields *n* = 35. Taking into account the factors such as loss of interviews, we will expand the sample by 30%, *n* = 46, and the sample needed for each of the two groups will be 46 cases. The eligible participants were randomly assigned to the control or intervention group using a random-number table by an independent statistician. To avoid mutual contamination between the two groups during hospitalization, the patients in the control and intervention groups were admitted to different wards. Blinding of the research objects was realized.

### 2.2 Measures

In addition to external factors such as marital status, occupation, education and medical expense, two instruments were used in this study to measure resilience and QOL.

#### 2.2.1 The Chinese version of the Connor–Davidson Resilience Scale

The participants’ resilience was assessed by the Chinese version of the Connor–Davidson Resilience Scale (CD-RISC). The scale consists of 25 items. It encompasses three subscales: tenacity, self-improvement, and optimism (18). Each item is rated on a five-point Likert scale (0 = never, 1 = seldom, 2 = sometimes, 3 = often, and 4 = routinely). The internal consistency reliability was reported as 0.91 for the total scale (19). Because the number of entries in each subscale is different, the factor score is used to make the scores of each subscale comparable. Factor score = mean score of subscale/number of entries. A higher score indicates a higher level of resilience. In this study, the internal consistency was shown by a Cronbach’s α of 0.861.

#### 2.2.2 The Chinese version of the 36-Item Short-Form Health Survey

The Chinese version of the 36-Item Short-Form Health Survey (SF-36), developed by Muldoon (20) and Wang (21), is a 36-item scale composed of eight subscales: vitality, mental health, physiological functions, overall health, physical pain, social functions, physiological functions, and emotional functions. The NRS-2002 has been widely used to evaluate QOL in China. The item scores were recoded and transformed to the range 0 (worst possible health state) to 100 (best possible health state) (22), and a higher score indicated better QOL. The Cronbach’s α reliability of the Chinese version of the SF-36 was 0.83, whereas it was 0.78 in this study.

### 2.3 Intervention and control conditions

After completing purpose-designed demographic questionnaires and baseline assessments, all consenting participants were randomized to receive either the empowerment-based intervention or routine care.

**Figure 1:**
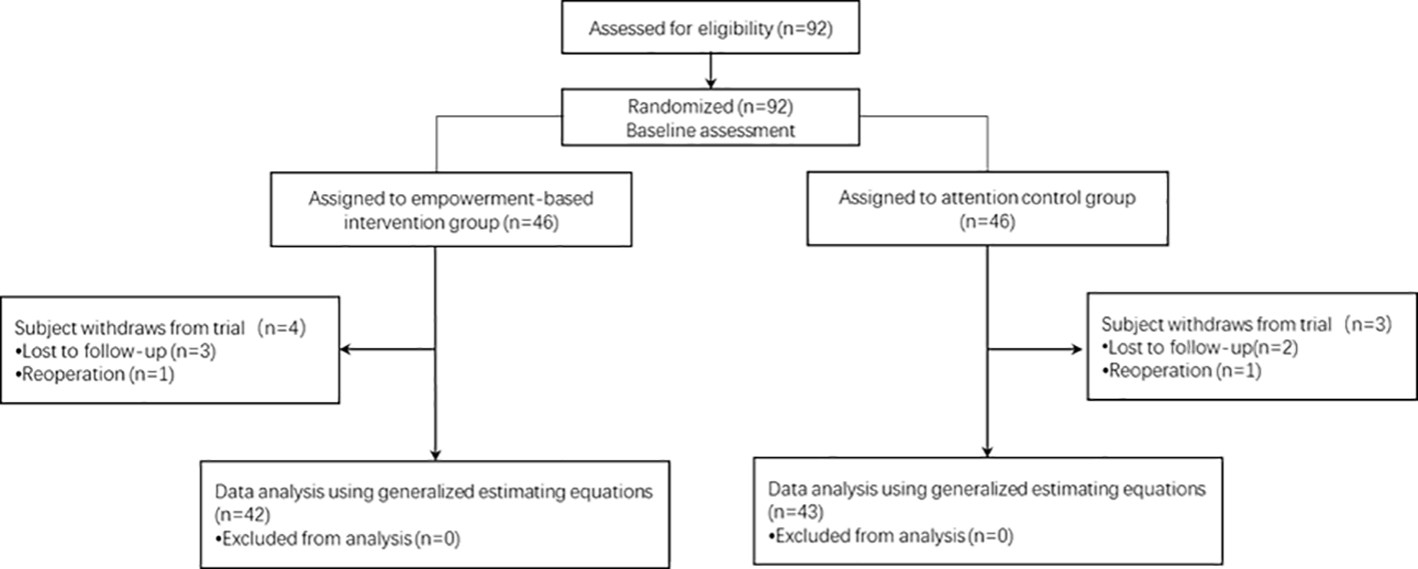
Cohort participant study flowchart. CONSORT, Consolidated Standards of Reporting Trials.

The age and sex of the participants lost to follow-up did not influence the homogeneity of the baseline demographic characteristics between the two groups. Finally, 42 participants in the intervention group and 43 participants in the control group completed the study. Participant recruitment, group assignment, and follow-ups are illustrated in **Figure 1**.

#### 2.3.1 Control group

Participants in the control group were followed up by telephone. After discharge, they received 4-weekly routine care comprising general health education and care provided by a cardiovascular nursing specialist.

#### 2.3.2 Telephone follow-up group (intervention group)

The participants in the intervention group underwent a telephone follow-up intervention based on empowerment in addition to routine care (see Section 2.3.1). The program was developed based on empowerment theory as a theoretical basis and framework of care. The intervention included five steps: defining problems, expressing emotions, setting goals, making plans, and evaluating results. Each intervention consisted of the five steps described above. The first intervention was performed on the day of discharge and once a week after discharge for 4 weeks; each intervention lasted for 20–30 min.

The five interventions implemented in the empowerment-based care program were as follows:

1. Recognize potential, clarify problems, and set goals.

On the discharge day, patients’ and their families’ doubts and difficulties related to disease-related knowledge and postoperative rehabilitation were assessed, and patients were encouraged to express their feelings and ideas. On this basis, the researcher explained the basic knowledge of the disease in detail, provided targeted answers to patients’ confusion, guided patients to realize the potential and importance of self-determination, and established short-term rehabilitation goals after discharge.

2. Patients should express their emotions, face them positively, and receive medication and emotional management guidance.

In the first week, consolidate and strengthen early intervention and dispel doubts. Guide patients to use drugs correctly and explain medication precautions and adverse drug reaction judgment and treatment methods. Patients were encouraged to express their feelings, learn self-regulation methods of negative emotions, clarify the problems existing in disease rehabilitation after discharge, and face them positively.

3. Build confidence, adhere to self-care, and give lifestyle guidance.

In the second week, consolidate and strengthen early intervention and dispel doubts. Guide patients to self-adjust their sleep, diet, exercise, etc., according to their need for disease rehabilitation. Encourage patients to reveal the problems existing in postoperative self-care and help them to face such problems positively, rebuild confidence, and maintain self-care.

4. Tap potential, strengthen self-care, and provide emergency self-help guidance.

In the third week, consolidate and strengthen early intervention and dispel doubts. With the continuous realization of this goal, patients can be guided to know the positive role of disease self-determination; fully tap patients’ potential, further mobilize their subjective initiative, guide patients to identify disease recurrence and acute complications in time, and effectively implement self-help.

5. Effect evaluation, adjustment and improvement, and encouragement of self-determination.

In the fourth week, patients were encouraged to consolidate and strengthen their early intervention, dispel doubts, and self-evaluate according to the goals set in the early stage; determine the reasons for the unfulfilled goals; make self-improvement plans; and continue to carry out disease self-determination and promote disease recovery.

### 2.4 Set up a research group

After determining the intervention plan, we set up the research team. (1) Management team: the head nurse of the cardiology department was responsible for coordinating the work of the implementation staff and controlling the quality of this study. (2) Professional team: after unified training, six nurses were responsible for the whole implementation of the intervention program. (3) Data analysis team: The researcher and a graduate student collected and analyzed the data.

Personnel of the management team and professional team participated in the whole process of intervention, because of the need for unified training before intervention; thus, blinding of the intervention personnel was not realized. The data analysis team was not involved in the intervention process and blinding of the data analysts was achieved.

### 2.5 Data collection methods

Collect the general information of patients in the medical record system, and verify the uncertain content face to face. Inform patients of the purpose and significance of this study before handing out the scale, to gain their trust and cooperation. Explain the purpose of the scale to patients one by one with the prescribed guidance, explain the precautions for filling in the questionnaire, ensure that the patient understands the questionnaire before filling it in and that the patient completes the questionnaire independently within 20 to 30 min, and check for omissions and duplications after retrieval and timely supplement. The daily survey data collected were reviewed on the same day, and the data were checked again after two people have independently inputted the data to ensure their accuracy.

### 2.6 Statistical analysis

Statistical analyses were performed using the software package SPSS 19.0. The chi-square test, Fisher’s exact probability method, and *t*-test were used for the equilibrium test, and the *t*-test and Mann–Whitney *U* test were used to explore the differences in intervention effects. The level of statistical significance was set at 0.05.

## 3 Results

### 3.1 Demographics

A total of 85 participants were enrolled in the study. **Table 1** shows the demographic information of the patients in the two groups. There were no significant differences between the groups in terms of age, sex, education level, number of surgeries, number of cardiac stents, cardiac function grade, resilience, or QOL.

**Table 1:** Demographic characteristics of the participants and resilience and quality of life scores at baseline.

Variables	Control (*n* = 43)	Intervention (*n* = 42)	*t*/*χ* ^2^	*p*
Age (years, $\bar{x}$ ± s)	60.86 ± 8.85	60.50 ± 8.90	0.187	0.852
Gender (*n*)			0.003	0.958
Male	35	34		
Female	8	8		
Level of education (*n*)			0.430	0.807
Primary school or below	7	9		
Junior and senior high school	25	22		
Junior college and above	11	11		
Number of operations (*n*)			1.120	0.571
1	31	26		
2	11	14		
≥3	1	2		
Number of cardiac stents (*n*)			1.826	0.401
1	16	10		
2	9	10		
≥3	18	22		
Cardiac function grade (*n*)			3.081	0.379
Normal	3	0		
Level I	6	7		
Level II	31	32		
Level III or above	3	3		
Resilience ( $\bar{x}$ ± s)				
Tenacity	31.63 ± 5.41	30.90 ± 5.66	0.602	0.549
Self- improvement	17.91 ± 4.11	17.71 ± 3.45	0.234	0.816
Optimism	7.33 ± 2.22	7.40 ± 2.12	−0.168	0.867
Total scores	56.86 ± 10.26	56.02 ± 8.96	0.400	0.690
Quality of life ( $\bar{x}$ ± s)				
GH	33.14 ± 13.89	32.98 ± 13.44	0.055	0.956
PF	71.63 ± 9.62	72.26 ± 10.43	−0.291	0.771
PRF	49.42 ± 14.93	49.40 ± 17.01	0.004	0.997
ERF	51.94 ± 22.19	48.41 ± 23.52	0.711	0.479
SF	49.13 ± 14.54	49.41 ± 16.09	−0.083	0.934
BP	53.92 ± 10.04	54.73 ± 12.15	−0.334	0.739
VT	63.37 ± 8.71	63.93 ± 9.60	−0.280	0.780
MH	65.67 ± 11.82	65.24 ± 10.40	0.181	0.857
Total scores	54.78 ± 6.39	54.54 ± 8.49	0.143	0.887

SF, social function; ERF, emotional role function; MH, mental health; VT, vitality; PF, physical function; PRF, physical role function; BP, bodily pain; GH, global health.

### 3.2 Resilience

**Table 2** shows a comparison of resilience. Compared to those in the control group, the total (*t* = −2.484 *p* = 0.015) and optimism (*t* = −4.252, *p* ≤ 0.001) scores improved significantly in the intervention group over time. Compared to those at T1, only the intervention group exhibited significant improvements in the total score and in the three domains of resilience at T2 (*t* = 3.974–6.184, *p* ≤ 0.001).

**Table 2:** Comparison of resilience.

Resilience	Control (*n* = 43)				Intervention (*n* = 42)				Between-group variation			
	Mean ± SD		Within-group variation		Mean ± SD		Within-group variation		T1 control vs. T1 intervention		T2 control vs. T2 intervention	
	T1	T2	*t*	*p*	T1	T2	*t*	*p*	*t*	*p*	*t*	*p*
Tenacity	31.63± 5.41	32.30 ± 6.94	0.678	0.502	30.90 ± 5.66	35.17 ± 7.50	3.974	<0.001**	0.542	0.590	−1.832	0.071
Self-improvement	17.91± 4.11	19.26± 5.35	1.764	0.085	17.71 ± 3.45	21.00 ± 4.88	6.169	<0.001**	0.243	0.809	−1.566	0.121
Optimism	7.33 ± 2.22	7.19 ± 1.96	−0.345	0.732	7.40 ± 2.12	9.62 ± 2.91	5.894	<0.001**	−0.149	0.882	−4.525	<0.001**
Total score	56.86± 10.26	58.74± 12.77	1.019	0.314	56.02 ± 8.96	65.79 ± 13.40	6.184	<0.001**	0.402	0.689	−2.484	< 0.05*

T1, at baseline; T2, at completion of the intervention.

*p < 0.05.

**p < 0.01 (two-tailed).

### 3.3 Quality of life

**Table 3** shows the SF-36 scores at T1 and T2. The patients in the intervention group had better global health (*t* = −2.659, *p* = 0.009), emotional role function (*t* = −2.516, *p* = 0.014), social function (SF, *t* = −2.256, *p* = 0.027), and total QOL than patients in the control group after a 1-month follow-up. Compared to those of the SF-36 at T1, the control group showed significant improvements in global health (*t* = 2.626, *p* = 0.012), social function (*t* = 2.108, *p* = 0.041), bodily pain (*t* = 4.321, *p* ≤ 0.001), vitality (*t* = −3.745, *p* = 0.001), mental health (*t* = 3.516, *p* ≤ 0.001), and total QOL (*t* = 3.826, *p* ≤ 0.001) at T2. Except for physical role function, the intervention group showed significant improvements in the other seven domains and total QOL after a month of follow-up.

**Table 3:** Comparison of quality of life.

Resilience and quality of life	Control (*n* = 43)				Intervention (*n* = 42)				Between-group variation			
	Mean ± SD		Within-group variation		Mean ± SD		Within-group variation		T1 control vs. T1 intervention		T2 control vs. T2 intervention	
	T1	T2	*T*	*p*	T1	T2	*t*	*p*	*t*	*p*	*t*	*p*
GH	33.14 ± 13.89	39.07 ± 13.01	2.626	0.012^*^	32.98 ± 13.44	49.29± 21.49	4.161	<0.001^**^	0.054	0.957	−2.659	0.009 ^**^
PF	71.63 ± 9.62	74.88 ± 13.82	1.577	0.122	72.26 ± 10.43	79.88± 16.14	3.041	0.004^**^	−0.290	0.773	−1.535	0.128
PRF	49.42 ± 14.93	51.16 ± 26.14	0.387	0.701	49.4 ± 17.01	56.55± 27.64	1.549	0.129	0.006	0.995	−0.924	0.358
ERF	51.94 ± 22.19	51.16 ± 21.02	−0.184	0.855	48.41 ± 23.52	65.08± 29.4	2.920	0.006^**^	3.590	<0.001^**^	−2.516	0.014 ^**^
SF	49.13 ± 14.54	56.11 ± 19.55	2.108	0.041^*^	49.4 ± 16.09	65.77± 19.92	5.518	<0.001^**^	−0.081	0.935	−2.256	0.027 ^**^
BP	53.92 ± 10.04	71.44 ± 24.56	4.321	<0.001^**^	54.73 ± 12.15	76.92± 18.83	7.462	<0.001^**^	−0.335	0.738	−1.152	0.252
VT	63.37 ± 8.71	69.77 ± 9.51	3.745	0.001^**^	63.93 ± 9.60	69.64± 14.75	2.671	0.011^*^	−0.282	0.779	0.048	0.962
MH	65.67 ± 11.82	72.47 ± 7.72	3.516	0.001^**^	65.24 ± 10.4	73.33± 14.83	3.806	<0.001^**^	0.178	0.860	−0.336	0.737
Total score	54.78 ± 6.39	60.76 ± 11.13	3.826	<0.001^**^	54.54 ± 8.49	67.06± 14.83	5.886	<0.001^**^	0.147	0.883	−2.219	0.029 ^**^

T1, at baseline; T2, at completion of the intervention.

*p < 0.05.

**p < 0.01 (two-tailed).

## 4 Discussion

To the best of our knowledge, this is one of the very few controlled trials to date to illustrate the application of an empowerment-based care program in patients with CHD. The findings of the present study indicated that, compared with participants in the control group, patients who received an empowerment-based intervention demonstrated significantly greater improvements in levels of resilience and QOL.

### 4.1 Effectiveness of the intervention on resilience

Better resilience can help patients establish confidence in their ability to overcome disease, improve their health status, promote their recovery from disease, and ultimately improve their QOL (23). The study showed that after 1 month of routine nursing follow-up after coronary stent implantation, the overall score of the control group improved by only 1.88 points, indicating that routine nursing is helpful for improving the patients’ psychological resilience. After a month of telephone follow-up based on the theory of empowerment, the patients’ resilience and scores on all dimensions were significantly greater in the intervention group than in the control group.

There are two main reasons for the effectiveness of this program. First, follow-up interventions based on the empowerment theory are different from routine nursing follow-up. It emphasizes patients in the dominant position. When providing knowledge support for patients, nurses should respect patients’ wishes, promote patients’ autonomy, and consider patients’ psychological needs. Second, the main purpose of the empowerment-based care program was to encourage patients to strengthen communication with their families and participate in social interaction. Therefore, the level of resilience of patients in the intervention group obviously improved.

### 4.2 Effectiveness of the intervention on quality of life

The current study showed that an empowerment-based care program could significantly improve the total QOL and QOL-8 status of patients with CHD. Compared to routine nursing, this intervention could improve patients’ GH, ERF, and SF to a greater extent. The effectiveness of empowerment-based care might result from several advantages, including individualized educational sessions based on a patient-centered comprehensive assessment, and continuous and repeated telephone follow-up counseling. Empowerment-based care can not only help patients develop personalized goals, and plan and achieve learning goals, but also motivate their initiative toward disease management. Participants were encouraged to find a healthy lifestyle with full consideration. Moreover, empowerment-based care significantly increased patients’ resilience. Resilience is considered to protect against the detrimental effects of perceived stress on cardiovascular and metabolic health (24). Therefore, such interventions may empower patients to play active and responsible roles in the process of disease recovery, which, in turn, leads to improved total QOL, especially GH, ERF, and SF, after coronary stent implantation.

It should also be mentioned that, in the present study, there was no significant difference between the control group and the intervention group in terms of PF, PRF, BP, VT, or MH. A possible explanation is that the improvements in patients’ physiological function, physical pain, vitality, and mental health are strongly influenced by therapeutic effects and individual differences, especially physiological function, physical function, and physical pain; moreover, short-term nursing interventions have difficulty achieving significant results. Therefore, a long-term empowerment-based telephone follow-up care program is needed, and more attention should be given to changes in patients’ physiological function, physical function, physical pain, vitality, and mental health, to further clarify their influencing factors and carry out targeted nursing interventions.

### 4.3 Limitations

This study has at least two limitations. First, participants in this study were recruited from a Grade-A tertiary hospital in Xi’an, which renders generalization difficult. Second, owing to the limitations of time and labor force, only 1 month of intervention was carried out in this study. Further studies with longer intervention durations and larger sample sizes of patients with CHD after coronary stent implantation are recommended to obtain a comprehensive empowerment theory model of this population.

## 5 Conclusion

Telephone follow-up based on the empowerment theory can effectively improve the resilience and QOL of patients after CASI. This follow-up approach can provide a theoretical basis and practical reference for hospitals and communities to carry out targeted continuing nursing for patients after CASI.

## Data availability statement

The raw data supporting the conclusions of this article will be made available by the authors, without undue reservation.

## Ethics statement

The studies involving humans were approved by Biomedical Ethics Committee of the Medical Department of Xi’an Jiaotong University. The studies were conducted in accordance with the local legislation and institutional requirements. The participants provided their written informed consent to participate in this study.

## Author contributions

YH: Writing – original draft, Conceptualization, Data curation. MW: Writing – original draft, Formal Analysis. LL: Investigation, Writing – review & editing. CG: Resources, Writing – review & editing. ZH: Resources, Writing – review & editing. YaL: Investigation, Writing – review & editing. YuL: Investigation, Writing – review & editing. YX: Visualization, Writing – review & editing. CN: Conceptualization, Funding acquisition, Supervision, Writing – review & editing. AL: Conceptualization, Project administration, Writing – review & editing.
