# Supplementary material for: Telephone follow-up based on empowerment theory to improve resilience and quality of life among patients after coronary artery stent implantation: a randomized controlled trial
Source: Front Psychiatry. 2024 Apr 25;15:1248424. doi: 10.3389/fpsyt.2024.1248424 (PMC11079610; doi:10.3389/fpsyt.2024.1248424)
Supplement: Supplementary file 1 [file DataSheet_1.pdf]

1 The intervention group received a telephone follow-up intervention based on empowerment theory

Patient-centered health education guided by empowerment theory was implemented. After discharge from the hospital, we provide patients with intensive education on knowledge and skills related to postoperative rehabilitation through telephone follow-up, and at the same time, we focus on guiding patients to discover and utilize their potential, assisting in the establishment of behavioral goals, supervising the implementation of patients' self-determination goals through telephone calls and solving the problems encountered by patients promptly, so that they can build up their confidence, give full play to their self-determination ability, and move from "other-help" to "self-help" to promote effective recovery. help" to "self-help", and promote effective postoperative recovery.

1.1 Intervention plan

The time, topic and content of telephone follow-up intervention based on empowerment theory are shown in Table 1.

Table 1 Telephone follow-up time, topic and content of the intervention group based on empowerment theory

Intervention time	Intervention topic	intervention content
First time (Day of discharge)	Recognize potential, clarify problems and set goals.	Evaluate patients' and their families' doubts and difficulties about disease-related knowledge and postoperative rehabilitation, encourage patients to express their feelings and thoughts, and on this basis, the researcher explains in detail the basic knowledge of the disease in the context of the patient's situation, and gives targeted answers to the patient's confusions, and guides the patient to recognize the potential of self-determination and its importance, and to set up short-term post-discharge rehabilitation goals.
Second time (First week after	Express emotions, face them positively	Consolidate and strengthen the pre-intervention, solve the confusion, guide the patients to correctly use drugs, explain the precautions of

discharge)	and give medication and emotional management guidance.	using drugs and the judgment and treatment of adverse drug reactions; encourage the patients to express their emotions, teach the methods of self-adjustment of bad emotions, clarify the problems of post-discharge rehabilitation, and encourage the patients to face them positively.
Third time (Second week after discharge)	Build confidence, adhere self-care and give lifestyle guidance	Consolidate and strengthen the pre-intervention and clarification, guide patients to make self-adjustment in sleep, diet and exercise according to the needs of disease recovery; encourage patients to tell the problems in postoperative self-care, guide them to face them positively to rebuild their confidence, and insist on self-determination of the disease.
Fourth time (Third week after discharge)	Tap potential, strengthen self-care and give emergency self-help guidance	Consolidate and strengthen the preliminary intervention and puzzle solving; with the continuous achievement of the goals, guide patients to recognize the positive role of disease self-determination, fully tap the potential of patients, further mobilize their subjective initiative, and guide patients to recognize the occurrence of disease recurrence and acute complications in time and effectively implement self-help.
Fifth time (Fourth week after discharge)	Effect evaluation, adjustment and improvement, and encourage self-determination	Consolidate and strengthen the preliminary intervention, solve the confusion, guide patients to conduct self-assessment according to the goals set up in the early stage, find out the reasons for not achieving the goals, formulate self-rectification plans, and encourage patients to continue to carry out self-determination of the disease, and promote the recovery of the disease.

During the follow-up, the intervention group adjusted the patient's program goals promptly according to the patient's primary care problem, ensuring that the patient's wishes were respected while the optimal care plan was developed and implemented. Before the start of each follow-up visit, the content of the previous follow-up visit was assessed, mainly including the mastery of knowledge related to coronary heart disease, the implementation of the program, and the evaluation of interventions for emerging problems. Questions were used to conduct the assessment, such as: (1) Do you take your medication as prescribed? If a patient has a poor memory and often forgets to take medication, the follow-up visit will focus on the knowledge of taking medication to strengthen the patient's understanding of the importance of taking medication as prescribed by the doctor and to realize the dangers of omitting medication. Through the development of a rehabilitation program, to help them find a good way to remember the memories such as rehabilitation diary smiley face paste or cell phone memo, etc., to promote the patient to achieve the goal of medication adherence. (2) Do you know what are the triggers of coronary heart disease? Are you aware of the triggers of coronary heart disease? (3) Do you know what symptoms should be taken seriously? What are the relevant first-aid measures? (4) Do you exercise now, about how many times a week? How many times a week? Are you concerned about the intensity of exercise? (5) Do you still smoke? How many cigarettes do you smoke per day, and have you reduced the number of cigarettes compared with the last time? (6) Has there been any improvement in the situation of sleeping time recently? How did you deal with it? (7) Have you insisted on measuring blood pressure, how was it measured, and what is your normal standard? According to the patient's answer, timely adjustment or development of targeted interventions.

1.2 Intervention content

According to the results of the first part of the present investigation study, combined with the actual clinical nursing work, after full justification, the design determined five aspects of the telephone follow-up intervention content based on empowerment theory.

(1) Basic knowledge of coronary heart disease

Educate patients to understand the concept of coronary heart disease, common risk factors, common clinical manifestations, common types, routine clinical examination, treatment and review notes.

(2) Drug knowledge

Instruct patients to take medication as prescribed by the doctor, not to stop, change

or adjust the dosage of medication without authorization, and not to omit medication. Inform patients of the use of drugs, side effects and precautions; if there is any discomfort, promptly inform the doctor in charge or go to the hospital for consultation.

(3) Lifestyle

Sleep: Instruct patients to drink less water before going to bed to avoid excessive urination at night, which may affect insomnia; avoid activities that stimulate the brain before going to bed; go to bed on time and make it a habit to soak feet in warm water before going to bed; massage feet to promote blood circulation; take slow deep breaths to keep the whole body relaxing; listen to soothing music or read books that recuperate the body and mind; it is advisable to adopt the head-high, foot-low, and right-lateral lying position for sleep. If the condition is serious and heart failure occurs, it is advisable to adopt the semi-recumbent position, avoiding lying on the left side or prone, to reduce respiratory difficulties. If necessary, take medicines for insomnia as prescribed by the doctor.

Diet: educate patients to quit smoking and alcohol; follow the principles of low-salt, low-fat, low-cholesterol and light diet. Avoid eating high-cholesterol food (crab, fish roe, egg yolk, animal offal, etc.), fried food, pickled food, spicy food, coffee, strong tea, and cold tea. Do not eat bananas on an empty stomach, do not eat too full, do not skip breakfast.

Rehabilitation Exercise: During hospitalization, patients and their families are taught the basic methods of rehabilitation exercise, such as bed limb activities, bedside activities, walking, etc. After discharge, patients are instructed to choose the appropriate exercise according to their rehabilitation, such as walking, jogging, cycling, tai chi, mountain climbing and other aerobic exercises. The frequency of exercise should be at least 3~5 times a week, 40~60 minutes each time, and can also be adjusted according to their recovery effect. The intensity of exercise is suitable for not experiencing panic and chest tightness. When you first start to exercise, you need to progress gradually, starting with small exercises, regular and continuous. The appropriate heart rate for exercise is [170-age (years)], and patients with a history of angina pectoris should exercise at a maximum heart rate of less than 110 beats per minute.

(4) Emotion management

Through talking with patients to understand their psychological status, encourage patients to express their inner feelings. Guide patients through psychological counseling and successful cases of surgery, help them relieve anxiety, depression and other negative emotions, and establish confidence to overcome the disease. Patients

who suffer from insomnia due to excessive pressure can be instructed to distract their attention and relieve pressure through neck and shoulder massage, light music therapy and self-relaxation. The education emphasizes the "seven taboos" of daily life emotion management precautions: avoid excessive laughter, avoid joyfulness, avoid anger and irritability, avoid nervousness and fear, avoid accidental shock, avoid mental tension, and avoid mental depression.

(5) Emergency first aid

Teach patients to recognize the aura of disease recurrence and acute complications in time, and effectively implement self-help. For example, if the aura of angina attack of coronary heart disease lasts for more than 20 minutes or symptoms such as dyspnea, pallor, cold sweat, confusion, fainting, or convulsions occur, all activities should be stopped immediately, rest on the spot, and 1 tablet of nitroglycerin should be taken under the tongue immediately. If the symptoms cannot be relieved after 5 minutes, take 1 tablet of nitroglycerin and 300mg of aspirin under the tongue again, and call the emergency center "120". Educate the patient's family members to accompany the patient frequently, urge the patient to change his/her lifestyle habits, take medication on time, and check his/her health regularly; emphasize the need to put emergency medicines within reach, and the need for family members to learn simple first-aid measures, such as cardiopulmonary resuscitation (CPR). Once the patient develops the above symptoms, closely observe the patient's condition and seek prompt medical attention.
